# Safety assessment of deutetrabenazine: real-world adverse event analysis from the FAERS database

**DOI:** 10.3389/fphar.2024.1498215

**Published:** 2024-12-23

**Authors:** Yanping Shu, Yuanhe Wang, Jiaoying Liu, Lingyan Hu, Sichao Tong, Gang Wu, Xianlin Zhu

**Affiliations:** ^1^ Department of Psychiatry of Women and Children, The Second People’s Hospital of Guizhou Province, Guiyang, China; ^2^ Department of Neurology, Huaining County People’s Hospital, Anqing, China; ^3^ Rehabilitation Department, The Second People’s Hospital of Guizhou Province, Guiyang, China; ^4^ Department of Acupuncture, Jinhua Wenrong Hospital, Jinhua, China; ^5^ Department of Clinical Psychology, The Third Affiliated Hospital of Soochow University, Changzhou, China

**Keywords:** deutetrabenazine, data analysis, FAERS, real-world, adverse events

## Abstract

**Background:**

Deutetrabenazine is a widely used drug for the treatment of tardive dyskinesia (TD), and post-marketing testing is important. There is a lack of real-world, large-sample safety studies of deutetrabenazine. In this study, a pharmacovigilance analysis of deutetrabenazine was performed based on the FDA Adverse Event Reporting System (FAERS) database to evaluate its relevant safety signals for clinical reference.

**Methods:**

Adverse events (AEs) of FAERS with deutetrabenazine as the primary suspect drug were collected from the first quarter (Q1) of 2017 to Q1 of 2024. Reporting Odds Ratio (ROR), Proportional Reporting Ratio (PRR), Bayesian Confidence Propagation Neural Network (BCPNN), and Empirical Bayesian Geometric Mean (EBGM) were used to mine AEs risk signals of deutetrabenazine. AEs were standardized and classified using the System Organ Class (SOC) and Preferred Terms (PTs) from Medical Dictionary for Regulatory Activities (MedDRA) version 23.0.

**Results:**

A total of 3,583 AEs with deutetrabenazine as the primary suspect drug were collected in this study. We found that these AEs involved 23 SOCs, and the positive signals were mainly concentrated in systemic disease and various reactions at the site of administration (n = 1816, ROR = 1.23, PRR = 1.18, IC = 0.24, EBGM = 1.18), neurological disorders (n = 1736, ROR = 3.02, PRR = 2.60, IC = 1.38, EBGM = 2.60) and psychiatric disorders (n = 1,659, ROR = 4.15, PRR = 3.52, IC = 1.82, EBGM = 3.52). We eventually identified 100 valid PTs that met the criteria of the four algorithms. Drug ineffective, dyskinesia, depression, somnolence, suicidal ideation were considered to be the common PTs of deutetrabenazine. Tongue thrust (n = 4, ROR 253.47, PRR 253.35, IC 7.88, EBGM 235.95), grunting (n = 5, ROR 78.49, PRR 78.45, IC 6.26, EBGM 76.71) and drooling (n = 17, ROR 13.21, PRR 13.19, IC 3.72, EBGM 13.14) were not mentioned in the specification, but the high signal intensity suggested that they may be the potential adverse reactions.

**Conclusion:**

The efficacy of deutetrabenazine may be accompanied by some potential adverse effects in several systems. Adverse events in psychiatric, neurologic, gastrointestinal and respiratory need to be monitored in clinical practice.

## 1 Introduction

Tardive Dyskinesia (TD) is a disorder in which the tongue, lower face, and jaw, as well as limbs (sometimes involving the muscles of the pharynx, diaphragm, or trunk) involuntarily spasms or dance-like movements occur after several months of use of neuroblocker drugs (Savitt and Jankovic, 2018; Caroff et al., 2020a). A prospective cohort study comprising 362 patients on long-term nerve blocking medications who did not present with TD at baseline revealed that the risk of TD development was 32% after 5 years of antipsychotic use, 57% after 15 years, and 68% after 25 years (Glazer et al., 1993). A meta-analysis comprising 41 studies revealed that the mean prevalence of TD in individuals with schizophrenia spectrum disorders was 25.3% (Carbon et al., 2017). TD has been demonstrated to have a significant impact on an individual’s daily activities and communication, causing distress and adversely affecting their quality of life and mental health. In some cases, it may even lead to suicide (Strassnig et al., 2018; Caroff et al., 2020b).

The precise mechanism by which TD occurs remains unclear. However, there is evidence to suggest that it may be related to hypersensitivity of dopamine receptors, as well as to hypofunction of the γ-aminobutyric acid (GABA) ergic system and oxidative stress (Bhidayasiri et al., 2013; Cho and Lee, 2013; Abe et al., 2023). Deutetrabenazine, a reversible inhibitor of vesicular monoamine transporter 2 (VMAT-2), has been demonstrated to reduce dopamine availability at hypersensitive D2 dopamine receptors, thereby improving involuntary movements. (Bhidayasiri et al., 2013; Vanegas-Arroyave et al., 2024). Deutetrabenazine was initially approved by the U.S. Food and Drug Administration (FDA) for the treatment of TD in 2017. Additionally, guidelines published by the American Academy of Neurology and Psychiatry include it as a class A recommendation for the treatment of TD (Bhidayasiri et al., 2018; Thomson et al., 2019; Keepers et al., 2020). Multiple clinical trials have confirmed the efficacy and safety of deutetrabenazine (Anderson et al., 2017; Fernandez et al., 2017; Fernandez et al., 2019). While improving TD symptoms, deutetrabenazine may also cause Adverse Events (AEs). Clinical trials often fail to detect some late-onset and rare adverse reactions, and safety information needs to be supplemented by post-marketing data analysis. Since its launch in 2017, deutetrabenazine has been widely used in the treatment of TD, but there is a lack of post-marketing safety studies with large samples.

Based on the data from the FDA Adverse Event Reporting System (FAERS), this study used multiple data mining techniques to analyze the AEs risk signals associated with deutetrabenazine, and to evaluate the potential risks of its use to provide clues for clinical safety.

## 2 Materials and methods

### 2.1 Data source

**FIGURE 1:**
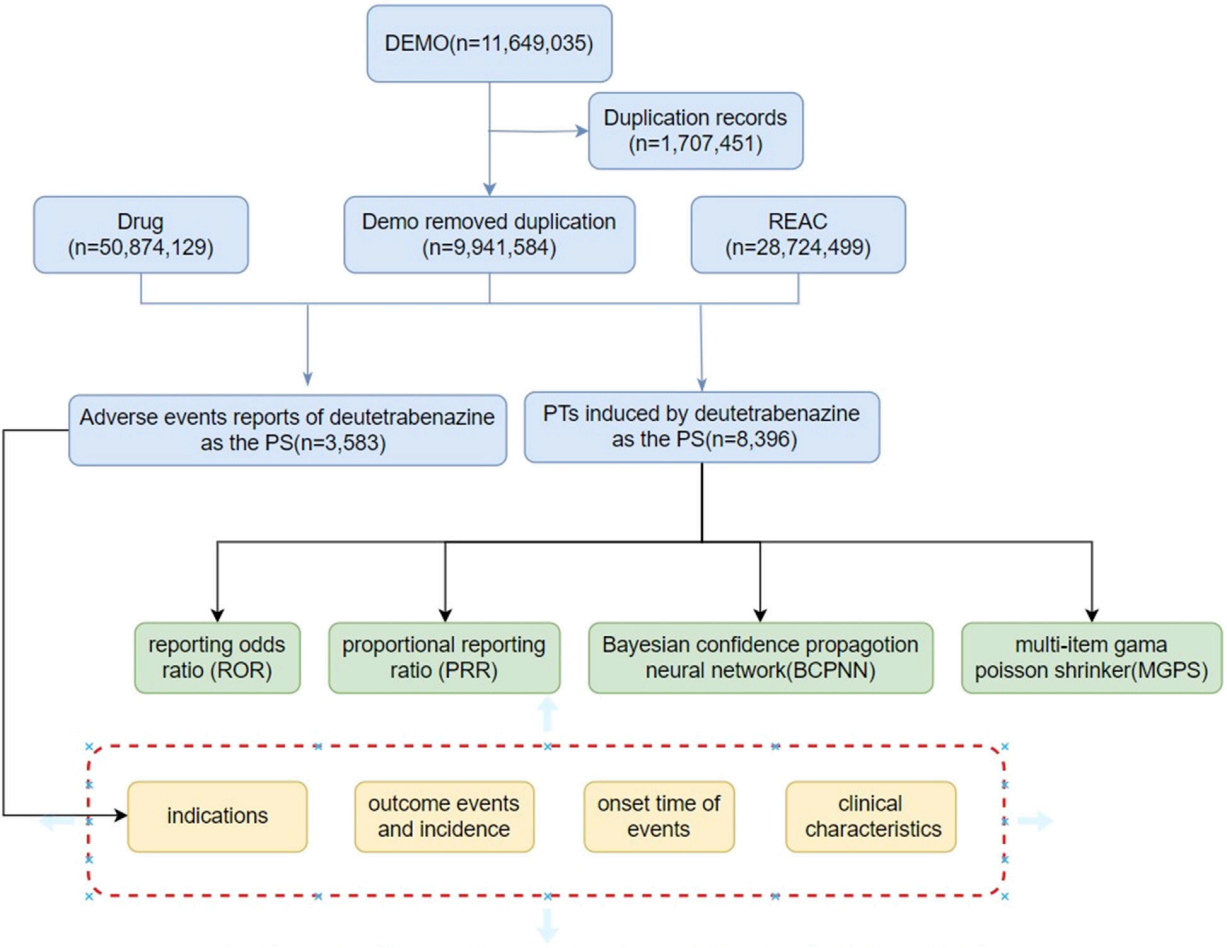
The Flow diagram of Selecting deutetrabenazine-related AEs from FAERS database.

The R4.4.1 software was employed to clean and initially analyze the American Standard Code for Information Interchange (ASCII) packets in the FAERS database from the first quarter of 2017 to the first quarter of 2024. The process is detailed in Figure 1. A total of 111,649,035 demographic records were removed from the Demographic and Administrative Information (DEMO) database, which contained a total of 111,649,035 demographic records, and 1,707,451 duplicate records were deleted. There were 28,724,499 data points in the reaction (REAC) table and 3,583 adverse reaction reports for deutetrabenazine.

### 2.2 Statistical analysis

In this study, the AEs of deutetrabenazine were assessed multidimensionally using four analytical methods. The main methods include the reporting odds ratio (ROR), proportional reporting ratio (PRR), Bayesian confidence propagation neural network (BCPNN) and empirical Bayesian geometric mean (EBGM) (Bate et al., 1998; DuMouchel, 1999; Evans et al., 2001; Rothman et al., 2004). Detecting and validating from multiple perspectives are more conducive to the accurate identification of safety signals. Among them, the ROR compares the ratio of target adverse events to non-target adverse events across the observed data. The PRR has better specificity than the ROR. Both the ROR and PRR calculations are based on 2 × 2 table, reflecting drug-event combinations, which can identify reports (Table 1) of AEs above the threshold, thereby highlighting the risk associated with deutetrabenazine and reducing the bias of lesser reported events. We set the number of reports (a) ≥ 3 and the lower limit of the 95% confidence interval (CI) > 1 as a positive signal in the ROR method. In the PRR method, a ≥3 and the lower limit of the 95% CI > 1 as a positive signal. BCPNN is mainly based on the information component (IC) and its 95% CI to evaluate drugs. BCPNN has the advantage of combining and cross-validating data from multiple sources. When the lower 95% CI of IC (IC025) is >0, it indicates a positive signal. Multi-item Gamma Poisson Shrinker (MGPS) is an extension of Gamma Poisson ShrinKer (GPS) to explore whether there is an association between medication population characteristics and adverse events and explore the interaction of variables. MGPS is used to calculate the empirical Bayesian geometric mean (EBGM), when the lower limit of the 95% CI of the EBGM (EBGM 05) is >2, it suggests a positive signal. Park and colleagues evaluated multiple approaches of data mining and found that there is no single method outperformed the others on all performance metrics, and they recommended using the common results of multiple methods to make decisions for adverse drug event surveillance (Park et al., 2020). Larger values indicate a stronger correlation between deutetrabenazine and AEs. The formulas are detailed in Table 2.

**TABLE 1:** Four grid table.

	Deutetrabenazine-related ADEs	Non-deutetrabenazine-related ADEs	Total
Deutetrabenazine	a	b	a+c
Non-deutetrabenazine	c	d	c + d
Total	a+c	b + d	N = a+b + c + d

**TABLE 2:** ROR, PRR, BCPNN, and EBGM methods, formulas, and thresholds.

Method	Formula	Threshold
ROR	$\text{ROR}=\frac{a\;/\;c}{b\;/\;d}$	a ≥3 ROR ≥3 95%CI (lower limit) > 1
	$SE\left(lnROR\right)=\sqrt{\frac{1}{a}+\frac{1}{b}+\frac{1}{c}+\frac{1}{d}}$	
	$95\%CI=e^{\ln\left(ROR\right)\pm 1.96se}$	
PRR	$\text{PRR}=\frac{a\;/\;\left(a+b\right)}{c\;/\;\left(c+d\right)}$	a ≥3 PRR ≥2 95%CI (lower limit) > 1
	$SE\left(lnPRR\right)=\sqrt{\frac{1}{a}-\frac{1}{a+b}+\frac{1}{c}-\frac{1}{c+d}}$	
	$95\%CI=e^{\ln\left(PRR\right)\pm 1.96se}$	
BCPNN	$\text{IC}=\log_{2}\frac{p\left(x,y\right)}{p\left(x\right)p\left(y\right)}=\log_{2}\frac{a\left(a+b+c+d\right)}{\left(a+b\right)\left(a+c\right)}$	IC025 > 0
	$E\left(\text{IC}\right)=\log_{2}\frac{\left(a+\gamma 11\right)\left(a+b+c+d+\alpha \right)\left(a+b+c+d+\beta \right)}{\left(a+b+c+d+\gamma \right)\left(a+b+\alpha 1\right)\left(a+c+\beta 1\right)}$	
	$V\left(\text{IC}\right)=\frac{1}{{\left(\ln2\right)}^{2}}\left[\frac{\left(a+b+c+d\right)-a+\gamma -\gamma 11}{\left(a+\gamma 11\right)\left(1+a+b+c+d+\gamma \right)}+\frac{\left(a+b+c+d\right)-\left(a+b\right)+a-\alpha 1}{\left(a+b+\alpha 1\right)\left(1+a+b+c+d+\alpha \right)}+\frac{\left(a+b+c+d+\alpha \right)-\left(a+c\right)+\beta -\beta 1}{\left(a+b+\beta 1\right)\left(1+a+b+c+d+\beta \right)}\right]$	
	$\gamma =\gamma 11\frac{\left(a+b+c+d+\alpha \right)\left(a+b+c+d+\beta \right)}{\left(a+b+\alpha 1\right)\left(a+c+\beta 1\right)}$	
	$\text{IC}-2\text{SD}=E\left(\text{IC}\right)-2\;\sqrt{V\left(\text{IC}\right)}$	
EBGM	$\text{EBGM}=\frac{a\left(a+b+c+d\right)}{\left(a+c\right)\left(a+b\right)}$	EBGM05 > 2
	$SE\left(lnEBGM\right)=\sqrt{\frac{1}{a}+\frac{1}{b}+\frac{1}{c}+\frac{1}{d}}$	
	$95\%CI=e^{\ln\left(EBGM\right)\pm 1.96se}$	

Abbreviations: 95% CI, 95% confidence interval; N, the number of reports; χ2, chi-squared; IC, information component; IC025, the lower limit of 95% CI, of the IC; EBGM, empirical Bayesian geometric mean; EBGM05, the lower limit of 95% CI, of EBGM.

### 2.3 Signal filtering and categorization

Drug names were standardized via the Medex_UIMA_1.8.3 system (Jiang et al., 2014). Preferred terms (PTs) of the AEs terminology set were standardized via the Medical Dictionary for Regulatory Activities (MedDRA) version 23.0 (Brown, 2004), and descriptions of the AEs classifications were standardized via the System Organ Class (SOC) (Tieu and Breder, 2018).

## 3 Results

### 3.1 Basic information of AEs reports

There were 3,583 AEs, with deutetrabenazine as the primary suspected drug, in the FAERS database from Q1 of 2017 to Q1 of 2024. The results revealed 89.51% of the reports included the sex of the patient, with the percentage of female patients (60.48%) being significantly greater than the percentage of male patients (29.03%). Approximately half of the reports (50.07%) did not provide information regarding the age of the patient, and among the reports with known ages, patients in the 45–65 age group were the most common, accounting for approximately 21.43%, followed by those in the 65–75 age group (13.76%). The number of reports of AEs associated with deutetrabenazine showed an annual increasing trend from 2017 to 2021 but a decreasing trend from 2021 to 2024. In addition, among the reports with noted AEs occurrences, the incidence of AEs was higher within 1 week of treatment and after 60 days of treatment. In terms of clinical outcomes, with the exception of unspecified serious outcomes, outcomes leading to hospitalization were the most common. Notably, the predominant reporting population in the report was consumers, who accounted for a greater proportion than healthcare professionals combined. Additionally, almost all reporting areas were from the United States, accounting for approximately 99.30% of the total. Detailed information is provided in Table 3.

**TABLE 3:** Basic information on AERs related to deutetrabenazine from the FAERS database.

Variable	Total
Year	
2017	49 (1.37)
2018	338 (9.43)
2019	577 (16.10)
2020	625 (17.44)
2021	776 (21.66)
2022	485 (13.54)
2023	483 (13.48)
2024	250 (6.98)
sex	
female	2,167 (60.48)
male	1,040 (29.03)
unknown	376 (10.49)
Age(years)	
<18	22 (0.61)
18–45	256 (7.14)
45–65	768 (21.43)
65–75	493 (13.76)
≥75	250 (6.98)
unknow	1794 (50.07)
Reporter	
Consumer	1967 (54.90)
Pharmacist	924 (25.79)
Physician	352 (9.82)
Other health-professional	173 (4.83)
unknown	167 (4.66)
Reported countries	
United States	3,558 (99.30)
other	25 (0.70)
Outcomes	
other serious	811 (56.40)
hospitalization	380 (26.43)
death	195 (13.56)
disability	23 (1.60)
life threatening	12 (0.83)
required intervention to Prevent Permanent Impairment/Damage	10 (0.70)
congenital anomaly	7 (0.49)
Adverse event occurrence time - medication date (days)	
<7	138 (6.52)
7–28	92 (4.35)
28–60	45 (2.13)
≥60	139 (6.57)
unknow	1703 (80.44)

### 3.2 Risk signal mining results

Table 4 describes the signaling intensity of deutetrabenazine at the SOC level. We found that the AEs associated with deutetrabenazine involved 23 SOCs. Positive signals were mainly concentrated in systemic diseases and various reactions at the site of administration (n = 1816, ROR = 1.23, PRR = 1.18, IC = 0.24, EBGM = 1.18), neurological disorders (n = 1736, ROR = 3.02, PRR = 2.60, IC = 1.38, EBGM = 2.60), psychiatric disorders (n = 1,659, ROR = 4.15, PRR = 3.52, IC = 1.82, EBGM = 3.52). All four algorithms for neurologic and psychiatric disorders had positive signals. In addition to the systems covered in the drug insert, some other systemic adverse reactions such as various musculoskeletal and connective tissue disorders, metabolic and nutritional disorders, and ophthalmic disorders were identified in this study.

**TABLE 4:** The signal strength of AEs of deutetrabenazine at the SOC level.

soc_English	Case reports	ROR (95% CI)	PRR (95% CI)	Chisq	IC(IC025)	EBGM (EBGM05)
psychiatric disorders	1659	4.15 (3.93, 4.38)	3.52 (3.38, 3.66)	3,175.65	1.82 (1.74)	3.52 (3.37)
nervous system disorders	1736	3.02 (2.87, 3.19)	2.6 (2.5, 2.7)	1862.09	1.38 (1.31)	2.6 (2.49)
congenital, familial and genetic disorders	30	1.24 (0.87, 1.78)	1.24 (0.87, 1.76)	1.4	0.31 (-0.2)	1.24 (0.92)
general disorders and administration site conditions	1816	1.23 (1.16, 1.29)	1.18 (1.13, 1.23)	59.76	0.24 (0.16)	1.18 (1.13)
injury, poisoning and procedural complications	883	0.87 (0.81, 0.93)	0.88 (0.83, 0.93)	15.62	−0.18 (-0.28)	0.88 (0.83)
gastrointestinal disorders	592	0.82 (0.76, 0.9)	0.84 (0.78, 0.91)	20.75	−0.26 (-0.38)	0.84 (0.78)
ear and labyrinth disorders	31	0.83 (0.58, 1.18)	0.83 (0.58, 1.18)	1.07	−0.27 (-0.77)	0.83 (0.62)
musculoskeletal and connective tissue disorders	288	0.63 (0.56, 0.71)	0.65 (0.58, 0.73)	59.36	−0.63 (-0.8)	0.65 (0.58)
respiratory, thoracic and mediastinal disorders	256	0.63 (0.56, 0.71)	0.64 (0.57, 0.72)	54.24	−0.64 (-0.82)	0.64 (0.58)
eye disorders	104	0.61 (0.5, 0.74)	0.62 (0.51, 0.75)	25.53	−0.7 (-0.98)	0.62 (0.52)
metabolism and nutrition disorders	104	0.59 (0.49, 0.72)	0.6 (0.49, 0.73)	29.08	−0.75 (-1.02)	0.6 (0.51)
cardiac disorders	106	0.59 (0.48, 0.71)	0.59 (0.48, 0.72)	30.35	−0.75 (-1.03)	0.59 (0.5)
investigations	264	0.5 (0.45, 0.57)	0.52 (0.46, 0.58)	124.62	−0.94 (-1.12)	0.52 (0.47)
reproductive system and breast disorders	22	0.37 (0.24, 0.56)	0.37 (0.25, 0.56)	23.94	−1.44 (-2.03)	0.37 (0.26)
vascular disorders	55	0.33 (0.25, 0.43)	0.33 (0.26, 0.43)	75.61	−1.59 (-1.97)	0.33 (0.27)
renal and urinary disorders	59	0.33 (0.25, 0.42)	0.33 (0.26, 0.43)	81.44	−1.59 (-1.96)	0.33 (0.27)
immune system disorders	35	0.32 (0.23, 0.45)	0.33 (0.24, 0.46)	49.38	−1.62 (-2.09)	0.33 (0.25)
infections and infestations	154	0.31 (0.26, 0.36)	0.32 (0.27, 0.37)	234.33	−1.64 (-1.87)	0.32 (0.28)
skin and subcutaneous tissue disorders	157	0.29 (0.25, 0.34)	0.31 (0.27, 0.36)	262.08	−1.71 (-1.93)	0.31 (0.27)
hepatobiliary disorders	20	0.27 (0.18, 0.42)	0.28 (0.18, 0.43)	38.41	−1.86 (-2.48)	0.28 (0.19)
endocrine disorders	5	0.22 (0.09, 0.52)	0.22 (0.09, 0.53)	14.23	−2.21 (-3.36)	0.22 (0.1)
neoplasms benign, malignant and unspecified (incl cysts and polyps)	14	0.05 (0.03, 0.08)	0.05 (0.03, 0.08)	275	−4.39 (-5.12)	0.05 (0.03)
blood and lymphatic system disorders	6	0.04 (0.02, 0.09)	0.04 (0.02, 0.09)	137.13	−4.61 (-5.68)	0.04 (0.02)

The present study identified 100 valid PTs that met the criteria of the four algorithms. Drug ineffectiveness, dyskinesia, depression, somnolence and suicidal ideation were considered common PTs of deutetrabenazine, and these common SOCs included general disorders and administration site conditions, nervous system disorders, and psychiatric disorders, as detailed in Table 5.

**TABLE 5:** The top thirty PTs of deutetrabenazine.

pt_English	n (%)
drug ineffective	513 (14.27)
dyskinesia	239 (6.65)
depression	238 (6.62)
somnolence	168 (4.67)
suicidal ideation	167 (4.65)
death	166 (4.62)
insomnia	163 (4.53)
tremor	163 (4.53)
fatigue	138 (3.84)
fall	131 (3.64)
dizziness	112 (3.12)
feeling abnormal	105 (2.92)
anxiety	103 (2.87)
off label use	103 (2.87)
product use in unapproved indication	100 (2.78)
gait disturbance	87 (2.42)
diarrhoea	80 (2.23)
nausea	80 (2.23)
confusional state	76 (2.11)
balance disorder	66 (1.84)
restlessness	66 (1.84)
agitation	65 (1.81)
headache	65 (1.81)
speech disorder	59 (1.64)
therapeutic product effect incomplete	59 (1.64)
dysphagia	58 (1.61)
wrong technique in product usage process	57 (1.59)
parkinsonism	57 (1.59)
dyspnoea	56 (1.56)
tardive dyskinesia	55 (1.53)

Table 6 presents the top 30 PTs that demonstrate compliance with all four algorithms. According to strict EBGM algorithm, PTs with higher risk signal strength includes tongue thrust (EBGM 235.95), grunting (EBGM 76.71), dyskinesia (EBGM 7.82), and drooling (EBGM 44.92). Comparison with the drug insert for deutetrabenazine revealed that tongue propulsion tongue thrust, grunting, and drooling were not mentioned in the insert and may be potential AEs.

**TABLE 6:** The top signal strength of AEs of deutetrabenazine ranked by EBGM at the PTs level.

soc_English	pt_English	Case reports	ROR (95% CI)	PRR (95% CI)	Chisq	IC (IC025)	EBGM (EBGM05)
nervous system disorders	chorea	21	105.58 (68.35, 163.07)	105.31 (68.42, 162.08)	2,105.04	6.68 (6.06)	102.2 (71.03)
nervous system disorders	dyskinesia	239	49.87 (43.81, 56.77)	48.48 (43.1, 54.53)	10,964.98	5.58 (5.39)	47.82 (42.91)
nervous system disorders	parkinsonism	57	47.9 (36.85, 62.27)	47.58 (36.88, 61.39)	2,564.19	5.55 (5.18)	46.94 (37.69)
nervous system disorders	drooling	41	45.72 (33.57, 62.26)	45.5 (33.25, 62.26)	1761.14	5.49 (5.05)	44.92 (34.69)
nervous system disorders	tardive dyskinesia	55	36.35 (27.85, 47.45)	36.12 (27.45, 47.52)	1858.82	5.16 (4.78)	35.75 (28.61)
nervous system disorders	facial spasm	5	34.78 (14.41, 83.95)	34.76 (14.39, 83.97)	162.29	5.11 (3.94)	34.42 (16.46)
nervous system disorders	oromandibular dystonia	3	33.43 (10.72, 104.26)	33.42 (10.72, 104.16)	93.44	5.05 (3.63)	33.11 (12.78)
nervous system disorders	parkinsonian gait	3	33.22 (10.65, 103.58)	33.21 (10.66, 103.51)	92.81	5.04 (3.62)	32.9 (12.7)
nervous system disorders	akathisia	41	24.83 (18.25, 33.79)	24.71 (18.06, 33.81)	926.43	4.62 (4.18)	24.54 (18.97)
nervous system disorders	essential tremor	3	23.93 (7.68, 74.49)	23.92 (7.67, 74.55)	65.42	4.57 (3.15)	23.76 (9.19)
nervous system disorders	tongue biting	5	23.44 (9.72, 56.5)	23.43 (9.7, 56.6)	106.62	4.54 (3.38)	23.27 (11.15)
nervous system disorders	head titubation	3	18.04 (5.8, 56.11)	18.03 (5.78, 56.2)	48.01	4.17 (2.75)	17.94 (6.94)
nervous system disorders	reduced facial expression	3	14.36 (4.62, 44.63)	14.35 (4.6, 44.73)	37.11	3.84 (2.42)	14.29 (5.53)
psychiatric disorders	soliloquy	6	50.21 (22.42, 112.45)	50.17 (22.46, 112.06)	284.99	5.63 (4.55)	49.46 (25.19)
psychiatric disorders	tic	26	45.39 (30.81, 66.88)	45.25 (30.58, 66.97)	1,110.58	5.48 (4.93)	44.68 (32.3)
psychiatric disorders	bruxism	23	35.45 (23.49, 53.49)	35.36 (23.43, 53.37)	760.02	5.13 (4.55)	35 (24.81)
psychiatric disorders	homicidal ideation	6	21.26 (9.52, 47.45)	21.24 (9.51, 47.44)	115.03	4.4 (3.33)	21.12 (10.79)
psychiatric disorders	suicidal ideation	167	16.24 (13.93, 18.94)	15.94 (13.63, 18.65)	2,330.74	3.99 (3.77)	15.87 (13.96)
psychiatric disorders	restlessness	66	14.45 (11.34, 18.42)	14.35 (11.34, 18.16)	816.58	3.84 (3.49)	14.29 (11.67)
gastrointestinal disorders	tongue thrust	4	253.47 (91.78, 700.03)	253.35 (91.43, 702.03)	936.08	7.88 (6.56)	235.95 (100.85)
gastrointestinal disorders	tongue movement disturbance	24	159.22 (105.69, 239.88)	158.77 (105.2, 239.62)	3,595.82	7.25 (6.67)	151.77 (107.71)
gastrointestinal disorders	salivary hypersecretion	17	13.21 (8.2, 21.28)	13.19 (8.24, 21.11)	190.77	3.72 (3.05)	13.14 (8.82)
respiratory, thoracic and mediastinal disorders	grunting	5	78.49 (32.34, 190.52)	78.45 (32.47, 189.51)	373.72	6.26 (5.09)	76.71 (36.53)
respiratory, thoracic and mediastinal disorders	nasal inflammation	4	24.71 (9.24, 66.08)	24.69 (9.27, 65.79)	90.29	4.62 (3.35)	24.52 (10.77)
injury, poisoning and procedural complications	drug titration error	15	31.22 (18.77, 51.93)	31.17 (18.72, 51.89)	434.08	4.95 (4.24)	30.9 (20.18)
injury, poisoning and procedural complications	drug dose titration not performed	6	18.79 (8.42, 41.92)	18.78 (8.41, 41.95)	100.42	4.22 (3.15)	18.68 (9.54)
eye disorders	excessive eye blinking	7	45.12 (21.4, 95.15)	45.09 (21.41, 94.96)	297.85	5.48 (4.47)	44.51 (23.85)
eye disorders	eyelid function disorder	3	36.66 (11.75, 114.37)	36.65 (11.76, 114.23)	102.92	5.18 (3.76)	36.27 (14)
musculoskeletal and connective tissue disorders	trismus	20	33.33 (21.45, 51.8)	33.25 (21.6, 51.18)	619.71	5.04 (4.42)	32.94 (22.78)
congenital, familial and genetic disorders	huntington’s disease	27	985.57 (642.09, 1,512.79)	982.4 (638.29, 1,512.02)	20,564.23	9.58 (8.98)	763.41 (533.39)

## 4 Discussion

There has been a gradual increase in adverse event reports for deutetrabenazine and a lack of comprehensive safety studies. To our knowledge, this is the first pharmacovigilance analysis of deutetrabenazine adverse events using FAERS data.

Our results revealed that female patients reported AEs at a higher rate than male patients did, and among reports with known ages, the 45–65 age group had the highest AEs incidence, followed by the 65–75 age group. It has been demonstrated that the risk of TD is elevated in older patients relative to younger patients (Sajatovic et al., 2022). This finding is consistent with the epidemiologic profile of TD, and previous studies have shown that female sex and advanced age are risk factors for TD (Solmi et al., 2018; Patterson-Lomba et al., 2019; Saklad, 2020). The metabolism and excretion of drugs are slowed in elderly patients, resulting in the accumulation of the drug in the body and an increased risk of adverse events (Akinosoglou et al., 2023). In light of the growing prevalence of deutetrabenazine, it is imperative for clinicians to remain vigilant for any adverse events associated with this medication, particularly in elderly female patients. The early detection of adverse events serves to reduce the risk of hospitalization and the risk of potentially developing life-threatening conditions. The impact of gender on deutetrabenazine-related adverse effects has yet to be fully elucidated, underscoring the need for further research in this area. It is noteworthy that the majority of reports in this category were submitted by consumers, representing a higher percentage of the total than other populations. This may have contributed to the lower accuracy of the reports. Consumer reports constitute an essential component of the FAERS (Munoz et al., 2019). Healthcare professional reports offer a more detailed clinical perspective, whereas consumer reports prioritize patient-related information and medication experience, both of which are crucial for ensuring the safe use of medications (Rolfes et al., 2015). Furthermore, the completeness of consumer reports is of value in comparison to those provided by healthcare professionals (Christ et al., 2023). The FDA ensures the quality of consumer reports through the implementation of relevant tools (Munoz et al., 2019). It would be beneficial to add further data on adverse reactions to deutetrabenazine in other countries in the future. In addition, we note that almost all reporting regions are from the United States. This may be related to factors such as database source, region, and time of drug launch. Deutertrabenazine was first approved by the FDA for the treatment of TD in the United States (Ricciardi et al., 2019).

Compared with tetrabenazine, deutetrabenazine contains deuterium, which reduces peak plasma concentrations to attenuate drug metabolism and reduces the frequency of administration, with fewer side effects (Claassen et al., 2017; Di-Martino et al., 2023). Both valbenazine and deutetrabenazine are approved by FDA for the treatment of delayed dyskinesia as VMAT-2 inhibitors. Metabolites of deutetrabenazine may act on the serotonin 5HT-7 receptor in addition to the VMAT-2 receptor. The occupancy of VMAT-2 receptor is 84% at low doses of valbenazine (40 mg), and 51%–92% at 6–42 mg doses of deutetrabenazine (Stahl, 2018). 2.5HT-7 receptor may play a role in regulating emotion (Kucwaj-Brysz et al., 2024). Deutetrabenazine induced parkinsonism symptoms are milder than those of valbenazine, which may be related to its receptor occupancy. It can better maintain the functional balance of dopaminergic neurons and reduce the severity of parkinsonism symptoms caused by excessive inhibition of the dopaminergic system (Zhang et al., 2024).

This study revealed that the risk signals for AEs caused by deutetrabenazine as the primary drug included 100 validated PTs in 23 SOCs. At the SOC level, these disorders mainly include general disorders and administration site conditions, nervous system disorders, and psychiatric disorders, which are consistent with the descriptions in the drug inserts. Adverse events associated with gastrointestinal, respiratory, thoracic and mediastinal disorders were also common. At the AEs level, the incidences of AEs such as dyskinesia, depression, somnolence, and suicidal ideation were high, again generally consistent with what was documented in the drug insert. Depression and suicidal ideation are frequently observed AEs. Although two randomized controlled trials have demonstrated the efficacy of deutetrabenazine in the treatment of tardive dyskinesia (Anderson et al., 2017; Fernandez et al., 2017), however, a meta-analysis by some scholars showed that deutetrabenazine 36 mg did not significantly improve TD symptoms, which may be due to insufficient therapeutic dose or poor quality of selected literature, resulting in selection bias (Ismail et al., 2024). To evaluate the efficacy of deutetrabenazine, it is necessary to consider not only the improvement of motor symptoms, but also psychological and social function, duration of action, and dose-response relationships. The black box warning of FDA suggests that deutetrabenazine may increase the risk of developing depression and suicidal ideation in patients with Huntington’s disease (Austedo, 2024). Dopamine plays a significant role in the etiology of depressive disorders, with the dopamine reward system implicated in the pathogenesis of depression (Wang et al., 2021; Murray et al., 2023). Some studies have demonstrated that the administration of pharmacological agents that enhance dopamine function can alleviate depressive symptoms (Delva and Stanwood, 2021; Mizuno et al., 2023). Deutetrabenazine exerts its pharmacological effects by inhibiting VMAT-2, which results in the inhibition of dopamine secretion into protruding vesicles and a reduction in overall dopamine release. This may potentially lead to an increase in depressive symptoms and suicidal ideation (Golsorkhi et al., 2024). In a study conducted by Schultz and colleagues, it is demonstrated that tetrabenazine do not elevate the likelihood of depression or suicidal ideation in patients diagnosed with Huntington’s disease. Among patients with a history of depression, those who used tetrabenazine exhibited a lower incidence of depression and suicidal ideation (Schultz et al., 2018). The study conducted by Frank and colleagues has demonstrated that deutetrabenazine has a lower incidence of TD compared to other treatments. The incidence of depression in Huntington’s disease patients treated with deutetrabenazine (4.8%) and a placebo (6.7%) was not statistically significant (Frank et al., 2024). The existing literature on the potential association between deutetrabenazine and depression and suicidal ideation is limited and inconclusive. To validate these findings, further large-scale prospective studies are required.

Furthermore, the study identified several novel PTs, including tongue thrust, grunting, and drooling. Deutetrabenazine, as a novel and highly selective VMAT-2 inhibitor, is a dopamine-depleting drug (Strassnig et al., 2018). The transport of neurotransmitters such as dopamine, epinephrine, and norepinephrine into synaptic vesicles (Guillot and Miller, 2009) reduced the concentration of dopamine, epinephrine, and norepinephrine in the protruding space and decreased the concentration of dopamine in the protruding space, affecting the oral muscle and causing tongue thrust. Deutetrabenazine depletes the stored neurotransmitters in the vesicles, reduces the concentration of the neurotransmitters, and the muscles of the tongue and throat relax excessively, thus blocking the airway and causing grunting (Guillot and Miller, 2009; Honda et al., 2020). Animal experiments have shown that the dopaminergic system can regulate saliva secretion, and deutetrabenazine may affect parotid salivation through VMAT, resulting in hypersalivation (Tomassoni et al., 2015).

Both our study and the drug insert indicate that deutetrabenazine is susceptible to neurologic and psychiatric adverse effects. Prevention and closely observation of neurologic and psychiatric symptoms are the primary methods of minimizing side effects (Bhidayasiri et al., 2013). Timely assessment, adjustment or discontinuation of dopamine receptor blockers, and quality nursing care are effective in mitigating adverse effects (Citrome et al., 2021). Some early-onset adverse events will subside after the therapeutic dose is reached. It is may be related to the therapeutic dose and tolerability (Frank et al., 2024). In addition, clinicians need to assess the patient’s condition in a timely manner. For the mild adverse reactions, we may continue to observe or treat symptomatically. However, for severe adverse reactions, discontinuation of medication and hospitalization are required (Stroup and Gray, 2018).

## 5 Limitations and future directions

Although this study evaluated the safety of deutetrabenazine in multiple dimensions, it is important to acknowledge that there are still some shortcomings. First, this study relied mainly on spontaneous reports and was mostly based on consumer reports, and the reported information may not be sufficiently comprehensive. Second, the reports in this database were mainly from the United States and lacked reports from other countries and regions, and there may be reporting bias. In the future, more rigorous prospective studies combining clinical trials and epidemiological studies are needed to assess the safety of deutetrabenazine more accurately and better guide its clinical use.

## 6 Conclusion

In conclusion, this study employed a multidimensional assessment of the safety of deutetrabenazine using the FAERS database. While some adverse reactions (e.g., tongue thrust, grunting, and drooling) manifest infrequently, they are notable indicators that warrant further investigation. In clinical practice, it is advisable to be mindful of the potential for adverse reactions in patients with psychiatric disorders, neurological disorders, gastrointestinal disorders, and respiratory conditions.

## Data Availability

The datasets presented in this study can be found in online repositories. The names of the repository/repositories and accession number(s) can be found below: www.fda.gov/drugs/drug-approvals-and-databases/fda-adverse-event-reporting-system-faers.

## References

[B1] Abe Y. Yagishita S. Sano H. Sugiura Y. Dantsuji M. Suzuki T. (2023). Shared GABA transmission pathology in dopamine agonist- and antagonist-induced dyskinesia. Cell. Rep. Med. 4, 101208. 10.1016/j.xcrm.2023.101208 37774703 PMC10591040

[B2] Akinosoglou K. Schinas G. Almyroudi M. P. Gogos C. Dimopoulos G. (2023). The impact of age on intensive care. Ageing Res. Rev. 84, 101832. 10.1016/j.arr.2022.101832 36565961 PMC9769029

[B3] Anderson K. E. Stamler D. Davis M. D. Factor S. A. Hauser R. A. Isojarvi J. (2017). Deutetrabenazine for treatment of involuntary movements in patients with tardive dyskinesia (AIM-TD): a double-blind, randomised, placebo-controlled, phase 3 trial. Lancet Psychiat 4, 595–604. 10.1016/S2215-0366(17)30236-5 28668671

[B4] Austedo (2024). Package insert. Teva neuroscience. Inc.

[B5] Bate A. Lindquist M. Edwards I. R. Olsson S. Orre R. Lansner A. (1998). A Bayesian neural network method for adverse drug reaction signal generation. Eur. J. Clin. Pharmacol. 54 (4), 315–321. 10.1007/s002280050466 9696956

[B6] Bhidayasiri R. Fahn S. Weiner W. J. Gronseth G. S. Sullivan K. L. Zesiewicz T. A. (2013). Evidence-based guideline: treatment of tardive syndromes: report of the guideline development subcommittee of the American Academy of Neurology. Neurology 81, 463–469. 10.1212/WNL.0b013e31829d86b6 23897874

[B7] Bhidayasiri R. Jitkritsadakul O. Friedman J. H. Fahn S. (2018). Updating the recommendations for treatment of tardive syndromes: a systematic review of new evidence and practical treatment algorithm. J. Neurological Sci. Official Bull. World Fed. Neurology. 389, 67–75. 10.1016/j.jns.2018.02.010 29454493

[B8] Brown E. G. (2004). Using MedDRA: implications for risk management. Drug Saf. 27 (8), 591–602. 10.2165/00002018-200427080-00010 15154830

[B9] Carbon M. Hsieh C. H. Kane J. M. Correll C. U. (2017). Tardive dyskinesia prevalence in the period of second-generation antipsychotic use: a meta-analysis. J. Clin. Psychiatry 78 (3), e264–e278. 10.4088/JCP.16r10832 28146614

[B10] Caroff S. N. Leong S. H. Roberts C. B. Berkowitz R. M. Campbell E. C. (2020a). Correlates of the abnormal involuntary movement scale in veterans with tardive dyskinesia. J. Clin. Psychopharmacol. 40 (4), 373–380. 10.1097/JCP.0000000000001229 32639290

[B11] Caroff S. N. Yeomans K. Lenderking W. R. Cutler A. J. Yonan C. Shalhoub H. (2020b). RE-KINECT: a prospective study of the presence and healthcare burden of tardive dyskinesia in clinical practice settings. J. Clin. Psychopharmacol. 40 (3), 259–268. 10.1097/JCP.0000000000001201 32332461 PMC7190052

[B12] Cho C. H. Lee H. J. (2013). Oxidative stress and tardive dyskinesia: pharmacogenetic evidence. Prog. Neuropsychopharmacol. Biol. Psychiatry 46, 207–213. 10.1016/j.pnpbp.2012.10.018 23123399

[B13] Christ P. Dubrall D. Schmid M. Sachs B. (2023). Comparative analysis of information provided in German adverse drug reaction reports sent by physicians, pharmacists and consumers. Drug Saf. 46, 1363–1379. 10.1007/s40264-023-01355-8 37987966 PMC10684666

[B14] Citrome L. Isaacson S. H. Larson D. Kremens D. (2021). Tardive dyskinesia in older persons taking antipsychotics. Neuropsychiatr. Dis. Treat. 17, 3127–3134. 10.2147/NDT.S328301 34703232 PMC8524363

[B15] Claassen D. O. Carroll B. De-Boer L. M. Wu E. Ayyagari R. Gandhi S. (2017). Indirect tolerability comparison of deutetrabenazine and tetrabenazine for huntington disease. J. Clin. Mov. Disord. 4, 3. 10.1186/s40734-017-0051-5 28265459 PMC5331691

[B16] Delva N. C. Stanwood G. D. (2021). Dysregulation of brain dopamine systems in major depressive disorder. Exp. Biol. Med. (Maywood) 246 (9), 1084–1093. 10.1177/1535370221991830 33593109 PMC8113739

[B17] Di-Martino R. Maxwell B. D. Pirali T. (2023). Deuterium in drug discovery: progress, opportunities and challenges. Nat. Rev. Drug Discov. 22, 562–584. 10.1038/s41573-023-00703-8 37277503 PMC10241557

[B18] DuMouchel W. (1999). Bayesian data mining in large frequency tables, with an application to the FDA spontaneous reporting system. Am. Statistician 53 (3), 177–190. 10.1080/00031305.1999.10474456

[B19] Evans S. J. Waller P. C. Davis S. (2001). Use of proportional reporting ratios (PRRs) for signal generation from spontaneous adverse drug reaction reports. Pharmacoepidemiol. and Drug Saf. 10 (6), 483–486. 10.1002/pds.677 11828828

[B20] Fernandez H. H. Factor S. A. Hauser R. A. Jimenez-Shahed J. Ondo W. G. Jarskog L. F. (2017). Randomized controlled trial of deutetrabenazine for tardive dyskinesia: the ARM-TD study. Neurology 88, 2003–2010. 10.1212/WNL.0000000000003960 28446646 PMC5440239

[B21] Fernandez H. H. Stamler D. Davis M. D. Factor S. A. Hauser R. A. Jimenez-Shahed J. (2019). Long-term safety and efficacy of deutetrabenazine for the treatment of tardive dyskinesia. J. Neurol. Neurosurg. Psychiatry 90, 1317–1323. 10.1136/jnnp-2018-319918 31296586 PMC6902058

[B22] Frank S. Anderson K. E. Fernandez H. H. Hauser R. A. Claassen D. O. Stamler D. (2024). Safety of deutetrabenazine for the treatment of tardive dyskinesia and chorea associated with huntington disease. Neurol. Ther. 13, 655–675. 10.1007/s40120-024-00600-1 38557959 PMC11136929

[B23] Glazer W. M. Morgenstern H. Doucette J. T. (1993). Predicting the long-term risk of tardive dyskinesia in outpatients maintained on neuroleptic medications. J. Clin. Psychiatry 54 (4), 133–139. 10.1016/0165-0327(93)90051-K 8098030

[B24] Golsorkhi M. Koch J. Pedouim F. Frei K. Bondariyan N. Dashtipour K. (2024). Comparative analysis of deutetrabenazine and valbenazine as VMAT2 inhibitors for tardive dyskinesia: a systematic review. Tremor Other Hyperkinet Mov. (N Y) 14, 13. 10.5334/tohm.842 38497033 PMC10941689

[B25] Guillot T. S. Miller G. W. (2009). Protective actions of the vesicular monoamine transporter 2 (VMAT2) in monoaminergic neurons. Mol. Neurobiol. 39 (2), 149–170. 10.1007/s12035-009-8059-y 19259829

[B26] Honda T. Takata Y. Cherasse Y. Mizuno S. Sugiyama F. Takahashi S. (2020). Ablation of ventral Midbrain/Pons GABA neurons induces mania-like behaviors with altered sleep homeostasis and dopamine D(2)R-mediated sleep reduction. iScience 23, 101240. 10.1016/j.isci.2020.101240 32563157 PMC7305386

[B27] Ismail O. Albdour K. Jaber Y. Jaber K. Alsaras A. (2024). Efficacy and safety of different pharmacological interventions in the treatment of tardive dyskinesia: a systematic review and network meta-analysis. Eur. J. Clin. Pharmacol. 80, 1471–1482. 10.1007/s00228-024-03722-5 38969949

[B28] Jiang M. Wu Y. Shah A. Priyanka P. Denny J. C. Xu H. (2014). Extracting and standardizing medication information in clinical text – the MedEx-UIMA system. Amia Summits Transl. Sci. Proc. 2014, 37–42. 25954575 PMC4419757

[B29] Keepers G. A. Fochtmann L. J. Anzia J. M. Benjamin S. Lyness J. M. Mojtabai R. (2020). The American psychiatric association practice guideline for the treatment of patients with schizophrenia. Am. J. psychiatry 177 (9), 868–872. 10.1176/appi.ajp.2020.177901 32867516

[B30] Kucwaj-Brysz K. Bas S. Zeslawska E. Podlewska S. Jastrzebska-Wiesek M. Partyka A. (2024). The importance of stereochemistry in 5-ht7r Modulation─A case study of hydantoin derivatives. Acs Chem. Neurosci. 15, 3884–3900. 10.1021/acschemneuro.4c00152 39433990 PMC11587507

[B31] Mizuno Y. Ashok A. H. Bhat B. B. Jauhar S. Howes O. D. (2023). Dopamine in major depressive disorder: a systematic review and meta-analysis of *in vivo* imaging studies. J. Psychopharmacol. 37 (11), 1058–1069. 10.1177/02698811231200881 37811803 PMC10647912

[B32] Munoz M. A. Delcher C. Dal-Pan G. J. Kortepeter C. M. Wu E. Wei Y. J. (2019). Impact of a new consumer form on the quantity and quality of adverse event reports submitted to the United States food and drug administration. Pharmacotherapy 39, 1042–1052. 10.1002/phar.2325 31479525

[B33] Murray L. Israel E. S. Balkind E. G. Pastro B. Lovell-Smith N. Lukas S. E. (2023). Multi-modal assessment of reward functioning in adolescent anhedonia. Psychol. Med. 53 (10), 4424–4433. 10.1017/S0033291722001222 35711146 PMC13345534

[B34] Park G. Jung H. Heo S. J. Jung I. (2020). Comparison of data mining methods for the signal detection of adverse drug events with a hierarchical structure in postmarketing surveillance. Life (Basel) 10, 138. 10.3390/life10080138 32764444 PMC7460123

[B35] Patterson-Lomba O. Ayyagari R. Carroll B. (2019). Risk assessment and prediction of TD incidence in psychiatric patients taking concomitant antipsychotics: a retrospective data analysis. BMC Neurol. 19 (1), 174. 10.1186/s12883-019-1385-4 31325958 PMC6642740

[B36] Ricciardi L. Pringsheim T. Barnes T. R. E. Martino D. Gardner D. Remington G. (2019). Treatment recommendations for tardive dyskinesia. Rev. Can. Psychiatr. 64 (6), 388–399. 10.1177/0706743719828968 PMC6591749 30791698

[B37] Rolfes L. van-Hunsel F. Wilkes S. van-Grootheest K. van-Puijenbroek E. (2015). Adverse drug reaction reports of patients and healthcare professionals-differences in reported information. Pharmacoepidemiol Drug Saf. 24, 152–158. 10.1002/pds.3687 25079444

[B38] Rothman K. J. Lanes S. Sacks S. T. (2004). The reporting odds ratio and its advantages over the proportional reporting ratio. Pharmacoepidemiol. Drug Saf. 13 (8), 519–523. 10.1002/pds.1001 15317031

[B39] Sajatovic M. Finkbeiner S. Wilhelm A. Barkay H. Chaijale N. Gross N. (2022). Long-Term safety and efficacy of deutetrabenazine in younger and older patients with tardive dyskinesia. Am. J. Geriatr. Psychiatry 30, 360–371. 10.1016/j.jagp.2021.08.003 34511333

[B40] Saklad S. R. (2020). Identifying tardive dyskinesia: risk factors, functional impact, and diagnostic tools. J. Clin. Psychiatry 81 (1), TV18059BR1C. 10.4088/JCP.TV18059BR1C 31944066

[B41] Savitt D. Jankovic J. (2018). Tardive syndromes. J. Neurological Sci. 389, 35–42. 10.1016/j.jns.2018.02.005 29506749

[B42] Schultz J. L. Killoran A. Nopoulos P. C. Chabal C. C. Moser D. J. Kamholz J. A. (2018). Evaluating depression and suicidality in tetrabenazine users with Huntington disease. Neurology 91, e202–e207. 10.1212/WNL.0000000000005817 29925548

[B43] Solmi M. Pigato G. Kane J. M. Correll C. U. (2018). Clinical risk factors for the development of tardive dyskinesia. J. neurological Sci. 389, 21–27. 10.1016/j.jns.2018.02.012 29439776

[B44] Stahl S. M. (2018). Comparing pharmacologic mechanism of action for the vesicular monoamine transporter 2 (VMAT2) inhibitors valbenazine and deutetrabenazine in treating tardive dyskinesia: does one have advantages over the other? CNS Spectr. 23, 239–247. 10.1017/S1092852918001219 30160230

[B45] Strassnig M. Rosenfeld A. Harvey P. D. (2018). Tardive dyskinesia: motor system impairments, cognition and everyday functioning. CNS spectrums 23 (6), 370–377. 10.1017/S1092852917000542 28877766

[B46] Stroup T. S. Gray N. (2018). Management of common adverse effects of antipsychotic medications. World Psychiatry 17, 341–356. 10.1002/wps.20567 30192094 PMC6127750

[B47] Thomson A. M. Wallace J. Kobylecki C. (2019). Tardive dyskinesia after drug withdrawal in two older adults: clinical features, complications and management. Geriatrics and Gerontology Int. 19 (6), 563–564. 10.1111/ggi.13669 31157524

[B48] Tieu C. Breder C. D. (2018). A critical evaluation of safety signal analysis using algorithmic standardised MedDRA queries. Drug Saf. 41 (12), 1375–1385. 10.1007/s40264-018-0706-7 30112728

[B49] Tomassoni D. Traini E. Mancini M. Bramanti V. Mahdi S. S. Amenta F. (2015). Dopamine, vesicular transporters, and dopamine receptor expression in rat major salivary glands. Am. J. Physiol. Regul. Integr. Comp. Physiol. 309, R585–R593. 10.1152/ajpregu.00455.2014 26136535

[B50] Vanegas-Arroyave N. Caroff S. N. Citrome L. Crasta J. McIntyre R. S. Meyer J. M. (2024). An Evidence-Based update on anticholinergic use for Drug-Induced movement disorders. Cns Drugs 38, 239–254. 10.1007/s40263-024-01078-z 38502289 PMC10980662

[B51] Wang S. Leri F. Rizvi S. J. (2021). Anhedonia as a central factor in depression: neural mechanisms revealed from preclinical to clinical evidence. Prog. Neuropsychopharmacol. Biol. Psychiatry 30 (110), 110289. 10.1016/j.pnpbp.2021.110289 33631251

[B52] Zhang Y. Jia X. Shi X. Chen Y. Xue M. Shen G. (2024). Mining of neurological adverse events associated with valbenazine: a post-marketing analysis based on FDA adverse event reporting system. Gen. Hosp. Psychiatry 90, 22–29. 10.1016/j.genhosppsych.2024.06.005 38901166

